# Early Changes in Crayfish Hemocyte Proteins after Injection with a β-1,3-glucan, Compared to Saline Injected and Naive Animals

**DOI:** 10.3390/ijms22126464

**Published:** 2021-06-16

**Authors:** Charlotta Ekblom, Kenneth Söderhäll, Irene Söderhäll

**Affiliations:** 1 Department of Comparative Physiology, Uppsala University, Norbyvägen 18 A, SE752 36 Uppsala, Sweden; charlotta.ekblom@ebc.uu.se; 2 Department of Comparative Physiology, Science for Life Laboratory, Uppsala University, Norbyvägen 18 A, SE752 36 Uppsala, Sweden; kenneth.soderhall@ebc.uu.se

**Keywords:** BGBP, clotting protein, crustacea, injection response, laminarin, proteome

## Abstract

Early changes in hemocyte proteins in freshwater crayfish *Pacifastacus leniusculus*, in response to an injection with the fungal pattern recognition protein β-1,3-glucan (laminarin) were investigated, as well as changes after saline (vehicle) injection and in naïve animals. Injection of saline resulted in rapid recruitment of granular hemocytes from surrounding tissues, whereas laminarin injection on the other hand induced an initial dramatic drop of hemocytes. At six hours after injection, the hemocyte populations therefore were of different composition. The results show that mature granular hemocytes increase in number after saline injection as indicated by the high abundance of proteins present in granular cell vesicles, such as a vitelline membrane outer layer protein 1 homolog, mannose-binding lectin, masquerade, crustin 1 and serine protease homolog 1. After injection with the β-1,3-glucan, only three proteins were enhanced in expression, in comparison with saline-injected animals and uninjected controls. All of them may be associated with immune responses, such as a new and previously undescribed Kazal proteinase inhibitor. One interesting observation was that the clotting protein was increased dramatically in most of the animals injected with laminarin. The number of significantly affected proteins was very few after a laminarin injection when compared to uninjected and saline-injected crayfish. This finding may demonstrate some problematic issues with gene and protein expression studies from other crustaceans receiving injections with pathogens or pattern recognition proteins. If no uninjected controls are included and no information about hemocyte count (total or differential) is given, expressions data for proteins or mRNAs are very difficult to properly interpret.

## 1. Introduction

Crayfish, as with other invertebrates, lack an adaptive immune system and are therefore reliant on their blood cells in response to pathogens or injury. In their natural environment, they are subjected to a number of potential pathogens, and their defense system needs to be fast and robust. The crayfish blood cells, called hemocytes, are the main effectors of the immune responses. They mediate cellular immunity by phagocytosis, encapsulation and hemocyte nodulation. The humoral responses act by melanization through the prophenoloxidase activating system (the proPO system), agglutination, and the release of antimicrobial peptides (AMPs) [1,2,3,4]. The immune system recognizes the invading pathogen through pattern recognition receptors (PRRs), many of which have been characterized in invertebrates. The cell wall components β-1,3-glucan and lipopolysaccharides (LPS) are recognized by the lipopolysaccharide and β-1,3-glucan binding proteins (LGBP), while β-1,3-glucans from fungal cell walls, also are recognized by the β-1,3-glucan recognition proteins (βGRPs). This will trigger the activation of immune responses [5,6,7,8,9,10]. The hemocytes of crayfish are the main effectors in these processes. These cells are divided into granular (GC), semi-granular (SGC) and hyaline (HC) cells, defined by morphological criteria [11,12], flow cytometry analysis [13,14], monoclonal antibodies [15,16,17] and protein markers [18,19]. The SGCs generally make up the largest part, while HCs make up a comparatively small part of total cells in crayfish. The HCs are capable of phagocytosis, while SGC are capable of encapsulation as well as phagocytosis to a lesser extent. Both SGC and GC, as the names suggest, contain granules with enzymes, AMPs, and components of the proPO system, which can be released into the extracellular space. Granular cells are the largest cells and contain a large number of granules, giving them their characteristic look [12]. The total number of hemocytes, as well as the cell type composition, varies considerably between individuals. After an infection or injury, the number of circulating hemocytes is rapidly depleted [20,21]. This leads to a need for new hemocytes to be released from the hematopoietic tissue (HPT), located as a sheet over the dorsal part of the stomach [22,23]. This process is regulated through different transcription factors, one of which is a RUNX family protein, and cytokine signaling [22]. In *Pacifastacus leniusculus* and other crustaceans, the astakines have been identified as being involved in this process [24].

A number of proteomic studies of crustacean hemocytes have been reported during recent years. Comparative proteomic profiles of crustacean hemocytes can be obtained with mass spectroscopy (MS) techniques, giving detailed information about the responses to infections and other stressors. A combination of 2-D electrophoresis and MALDI-TOF/TOF MS analysis was used to identify differential protein profiles of *Litopenaeus vannamei* hemocytes subjected to cold stress [25]. Six proteins were detected as upregulated after cold treatment for 24 h, and for some of these, an increase in hemocyte mRNA could also be detected [25]. Other studies have focused on proteome changes during different diseases such as white spot syndrome virus (WSSV), *Vibrio alginolyticus*, and *Spiroplasma* infections [26,27,28,29,30]. However, it is difficult to draw any solid conclusions from several of these studies, perhaps due to differences in experimental approach. A combination of 2-D electrophoresis and LC-MS/MS analysis of two different crustacean species, *Scylla olivacea* [27], and *Fenneropenaeus chinensis* [26] did not give similar results. A later study of proteomic changes after WSSV infection in the related mud crab *Scylla paramamosain*, in which a total proteome analysis by LC-ESI-MS/MS analysis was performed, did not confirm the earlier data from *S. olivacea*. For example, these studies reported opposite results for the anti-lipopolysaccharide factor 5 [26,28]. Different results in proteomic analysis were also obtained by Hou et al. [29,30], after infection with *Spiroplasma eriocheiris* in *Macrobrachium rosenbergii* [29] and *Eriocheir sinensis* [30], respectively. Moreover, another obstacle to obtain comparable results is the lack of genomic data for all the different species, causing difficulties in accessing gene sequences (or mRNAs) corresponding to the detected peptide fragments. In particular, the response to LPS has been investigated in several studies and has revealed important effectors in the infection response [18,20,31,32,33,34].

The ability to separate and identify hemocyte subpopulations through the use of density centrifugation in Percoll, and the use of hemocyte-specific monoclonal antibodies, has made it possible to learn more about hemocyte differentiation, and find molecular markers for the different cell types [11,16,17]. In 2019, our group performed a proteomic analysis of the profiles of GC, SGC, and cells of the hematopoietic tissue (HPT) and anterior proliferation center in the HPT of *P. leniusculus* [19]. Using a transcriptome database (Bioproject: PRJNA259594) for searches, this revealed some new markers in hemocyte lineage development, as well as new cell type-specific proteins [19]. However, still these markers are linked to one of two hemocyte types categorized by density centrifugation, and it is likely that each of these populations is a mix of different subtypes.

Work to better understand the crustacean response to fungal pathogens is still an ongoing process. In the present study, we focus on early changes in proteins in the circulating hemocytes of this freshwater crayfish, after an injection with laminarin, a β-1,3-glucan, in order to find some specific proteins of interest for host-fungus interaction. An important issue to address when trying to get an idea of a specific immune response after injection experiments is the fact that the composition of the hemocyte population may change dramatically upon any injection. Therefore, it is of high importance to understand how the hemocyte composition in the circulation is affected by a challenge with pathogens or pathogen-associated molecules, as well as any control injection as the responses may be very different. This issue is further addressed in the present study.

## 2. Results

### 2.1. Differential Cell Counts

**Figure 1:**
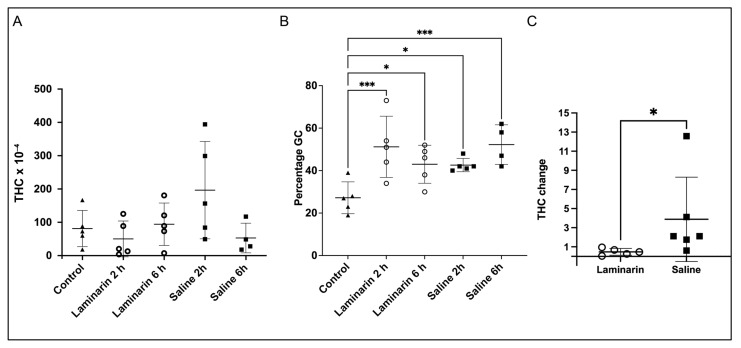
Total and differential hemocyte number in the circulation after saline or laminarin injection. (**A**) Total hemocyte count (THC) (cells per mL) in non-injected crayfish (control) and at two or six hours after injection of crayfish saline or laminarin (5 mg/mL) respectively. (**B**) Percentage of granular hemocytes in non-injected crayfish (control) and at two or six hours after injection of crayfish saline or laminarin (5 mg/mL), respectively. Line and error bars represent mean and standard deviations. Statistical analyses (were performed with one-way ANOVA (with Fisher LSD test) and asterisks * *p*-value < 0.05, *** *p*-value < 0.001 indicate significant differences compared to the control group. (**C**) Changes in total hemocyte number in the circulation after saline or laminarin injection. Each dot represents the relative hemocyte number in one individual crayfish six hours after injection compared to the number before injection in the same animal. The value one (1) in figure C means that no change occurred. Statistical analyses were performed with a Mann–Whitney test, and one asterisk (*) means *p*-value < 0.05, indicating significant differences between laminarin and saline injections.

Hemocyte numbers in circulation vary a lot in crayfish, and also after injection of laminarin or saline there is a great variation in the total number, as is shown in Figure 1a. Even so, injection of laminarin, as well as saline, led to a significantly higher proportion of granular hemocytes (Figure 1b). Due to the high individual variability in total hemocyte count (THC) in different animals, effects of the injections were investigated in each individual before and after injections with either saline or laminarin. Thus, hemocyte samples were taken from each individual crayfish and then each crayfish was allowed to rest for three days before the injections with laminarin or saline were performed. Six hours after injection a hemocyte sample was withdrawn from each individual animal and THC determined. As shown in Figure 1c, laminarin induced a significant decrease in THC, while saline injection resulted in that THC increased to a variable extent.

**Figure 2:**
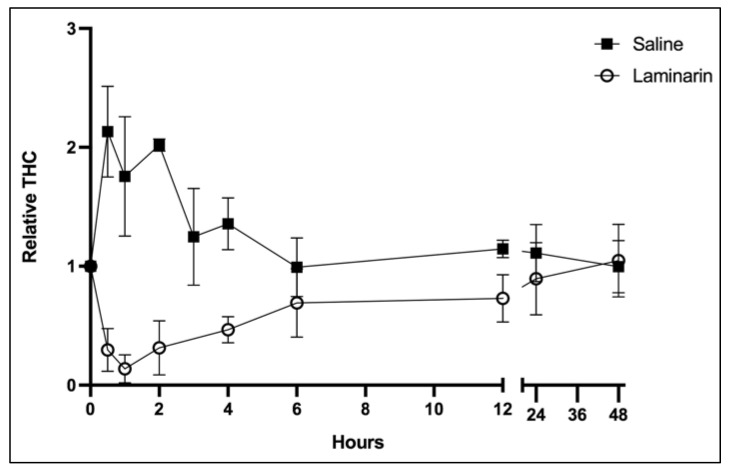
Changes in total hemocyte number (THC) freely circulating after saline or laminarin injection relative to values before injection. Each dot represents the mean and standard deviation and the number of animals at each time point (N) were 6–14.

We know from earlier studies that injection of saline (as well as PBS or other sterile isotonic solutions) results in rapid recruitment of hemocytes from tissues and thus a high increase in total number in the circulation, whereas injection of pathogens, LPS or β1,3-glucans leads to an initial dramatic drop in hemocyte number. This has to be considered for a further analysis of gene as well as protein expression. In Figure 2, we show that 80–90% of the peripheral hemocytes are lost after laminarin injection, whereas saline injection results in a rapid increase in THC. Although the original number of hemocytes varies considerably between individual crayfish the pattern is clear and always similar for these treatments. After the initial drop in hemocytes in laminarin injected animals, and an increase in THC in saline-injected ones there is a gradual restoration to the initial values after 24–48 h (Figure 2). This is well known for crustaceans and has to be considered when analyzing expression data.

### 2.2. Proteomic Analysis

From Figure 2 it is evident that a hemocyte population at six hours after injection of saline or laminarin (dissolved in saline) may be the result of two different processes. According to earlier studies injections of saline or other solutions without any pathogen-associated molecules result in rapid recruitment of hemocytes from different tissues, whether the recovery of hemocytes after injection of laminarin is mainly caused by release from the hematopoietic tissue [22,35]. To test if a global proteomic analysis could be used to detect any early protein markers for the crayfish response to a fungal infection, and to find possible marker proteins to newly released cells from the HPT, laminarin, a β-1,3-glucan, was injected into the animals, and hemocytes were collected after six hours. As controls, uninjected animals, as well as saline injected animals were used. We first had to understand changes in proteome occurring after a saline challenge. Thus, we analyzed the proteome of collected hemocytes from saline-injected crayfish after six hours and compared to uninjected animals. In total, the global proteome of five individuals per treatment was analyzed, and we used the *P. leniusculus* transcriptome database to identify the proteins as described in material and methods and all resulting data are presented in Supplementary Table S1.

**Figure 3:**
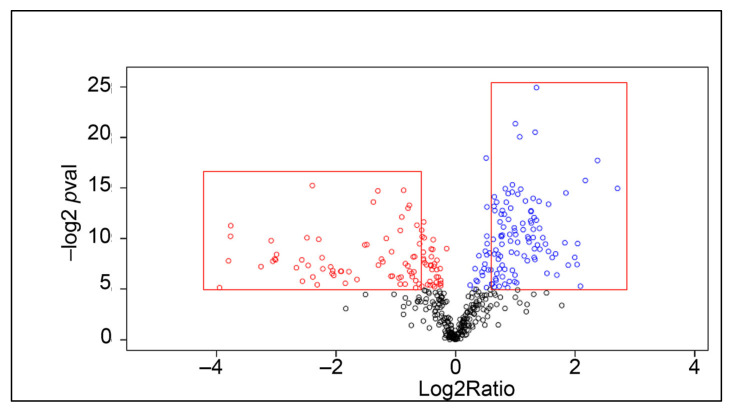
Differentially expressed proteins. Volcano plot showing differentially expressed proteins in hemocytes six h after saline injection. Significantly higher expressed proteins are shown in blue color and lower expressed proteins are shown in red color. The red frames indicate significant according to our criteria (Log2Ratio > 0.58, −log2 *p* value > 5).

When proteins that were present in all samples were compared, as many as 104 proteins were increased in amount after injection with saline (limit fold change > 1.5, Log2ratio > 0.58). The result of this first analysis is displayed in the volcano plot in Figure 3, and a list of these proteins is provided in Supplementary Table S2. The twelve most upregulated proteins are shown in Table 1 below.

To uncover proteins which may be of biological relevance for the treatment, hits that were both abundant and high in fold change after any treatment injection were detected using GiaPronto and are named “biomarkers.” For saline injections such biomarker proteins are presented in Table 2.

It is striking that all the six most abundant and highly upregulated proteins according to Table 2, except for GPX, are earlier shown to be characteristic of granular content in GC, and could be released by addition of a Ca^2+^-ionophore [36]. Moreover, it is well known that *P. leniusculus* expresses a high number of different Kazal proteinase inhibitors, which are secreted proteins, and are also present in granules of GC [37,38]. Thus, it seems clear from Table 2 as well as Figure 1b that saline injection will result in a rapid recruitment of GC, most likely from infiltrated GC in different tissues in the animals.

**Figure 4:**
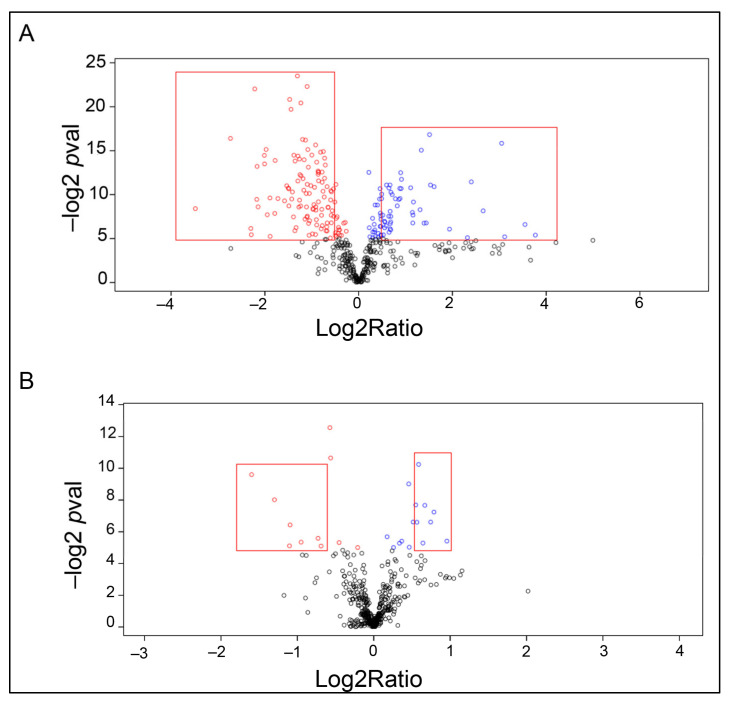
Differentially expressed proteins after laminarin injection. Volcano plot showing differentially expressed proteins in hemocytes six h after laminarin injection compared to saline (**A**) and to uninjected crayfish (**B**). Significantly higher expressed proteins are shown in blue color and lower expressed proteins are shown in red color. The red frames indicate significant according to our criteria (Log2Ratio > 0.58, –log2 *p* value > 5).

The dramatic effect on protein expression in the hemocyte population after a saline injection due to an increase in certain cell types means that it can be problematic to find proteins that are specifically upregulated after an injection with a pathogen molecule such as laminarin or LPS. Therefore, we used uninjected crayfish as double controls and investigated the differences in proteins between laminarin (dissolved in saline) with saline-injected as well as uninjected animals. When laminarin injected crayfish were compared to saline-injected ones, we found 41 proteins that were upregulated, while a comparison with uninjected animals only revealed six significantly upregulated proteins. These results are presented in Figure 4a,b. Since we had reason to believe that the cell populations examined in the three cases are very different and that the hemocytes freely circulating in the circulation six hours after an injection with saline or laminarin come from different sources in the animal (Figure 2), we further examined in detail the proteins that were upregulated and downregulated after injection with laminarin compared to saline (Figure 4). We could then observe that most, except a few, of the proteins that were significantly upregulated in laminarin compared to saline, were also downregulated in saline-injected crayfish, compared to uninjected animals. Similarly, most upregulated proteins in saline-injected crayfish compared to uninjected (Figure 3) were downregulated in the laminarin-injected (Supplementary Tables S2 and S3). Again, this result indicates that the composition of hemocyte types is very different in the different treatments, and that the proteomic analysis more or less reflects very diverse cell types more than changes in expression within the hemocytes. However, a few proteins were detected as specifically upregulated in laminarin compared to saline, and in laminarin compared to uninjected crayfish, and could indicate a response to β-1,3,-glucan (Table 3), while a few other proteins were upregulated both after laminarin and saline injection and could possibly be markers of a general wounding or stress response (Table 3).

Three proteins were clearly upregulated after saline as well as laminarin injection, one lysozyme, one crustin antimicrobial peptide and masquerade [39,40], which may indicate that these proteins are regulated as a general response to injury (Table 3). When laminarin injected crayfish were compared to uninjected controls, three proteins appeared upregulated which were not found in the saline-injected animals. These proteins were: a glycine-rich peptide, a Kazal proteinase inhibitor, and one putative chitin-binding protein (none of these are previously described). Two other proteins, the clotting protein and alpha actinin were detected with the highest fold change in laminarin-injected compared to uninjected animals (log2ration 2.02 and 1.15 respectively), but with higher –log2pvalue (2.3 and 3.5), and also detected as highly upregulated when laminarin was compared to saline injection (log2ration 3.67 and 1.93; –log2pvalues 2.5 and 4.5, respectively.

**Figure 5:**
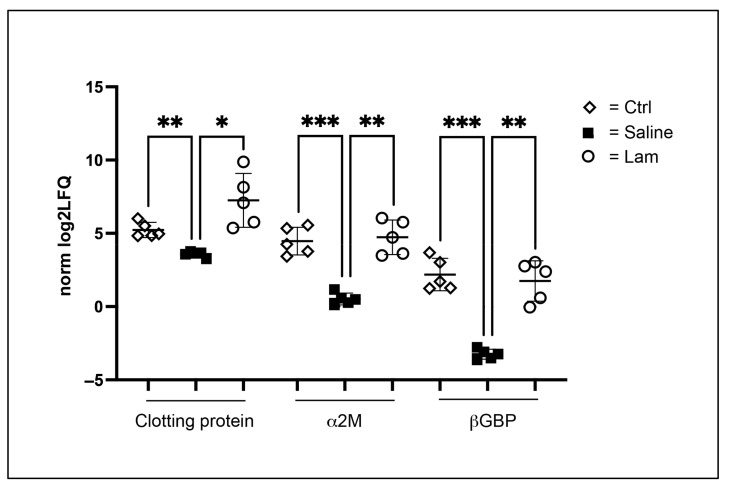
Highly upregulated plasma proteins after laminarin injection compared to saline injection. Statistical analyses were performed with a one-way ANOVA test, *p*-value < 0.0332 (*), <0.0021 (**) and <0.0001 (***).

An interesting observation was the biomarkers detected after laminarin compared to saline injections. The most abundant and highly upregulated proteins after laminarin injection were clotting protein, alpha-2-macroglobulin (α2M) and beta-1,3-glucan binding protein (βGBP) (Figure 5). Among these, the clotting protein was also increased compared to the uninjected controls. All these proteins are present in high amounts in plasma [5,41,42]. This means that they are most likely attached to the outside of some of the hemocytes, and at least for βGBP a 350 kDa membrane receptor has been purified from isolated hemocyte membranes, showing that binding of this protein to hemocytes occurs [6].

### 2.3. mRNA Expression

**Figure 6:**
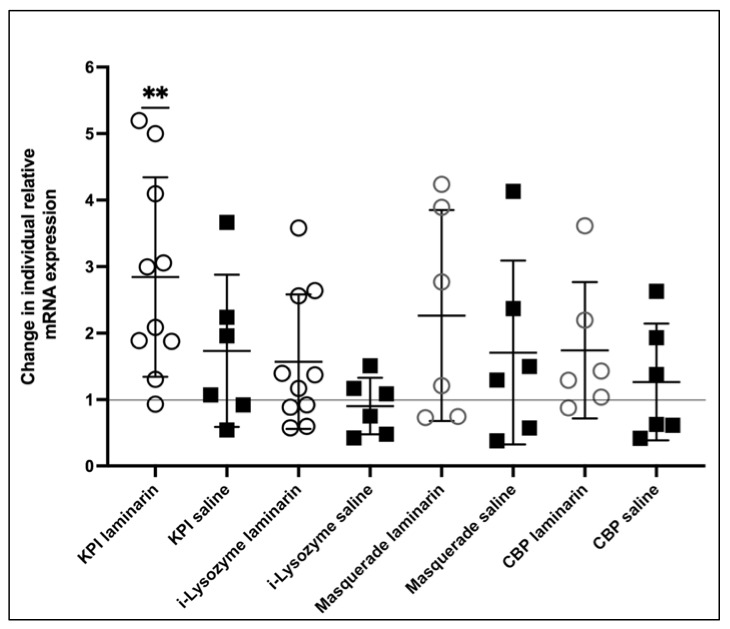
Changes in relative mRNA expression after saline or laminarin injection. Each dot represents the change in expression in hemocytes from one individual animal, six hours after injection. Statistical analyses were performed with a One sample t- and Wilcoxon test where two asterisks (**) means a *p*-value < 0.005, indicating significant change in expression after injection compared to the expression before. KPI: Kazal-type protease inhibitor, CBP: putative chitin-binding protein.

A semi-quantitative mRNA expression analysis of the upregulated proteins in Table 3, except for the glycine-rich peptide were performed at six h post-injection, using reverse transcription PCR. These genes were found to be constitutively expressed in all samples, with some individual variation. (Primers specific enough for the glycine-rich peptide could not be designed since we found out that there are several similar sequences present with small variation.) No clear difference in expression was detected between the different groups two hours or six hours after laminarin injection (data not shown). Due to considerable individual variation among samples, we decided to investigate mRNA expression in each individual before and after injection with either laminarin or saline. First, we analyzed the relative expression by semi-quantitative RT-PCR which revealed putative changes in expression of the Kazal proteinase inhibitor, the i-type lysozyme, masquerade and the putative chitin-binding protein. Therefore, we analyzed the samples further by quantitative reverse transcription PCR (qRT-PCR). The results presented in Figure 5 shows that laminarin injection resulted in a significant increase in mRNA expression of the Kazal proteinase inhibitor, while saline did not. Furthermore, no significant increase in expression of the other three transcripts could be detected at this time point (Figure 6).

## 3. Discussion

In the present study, the global proteome in hemocytes of healthy male *P. leniusculus* crayfish was investigated at six hours after injection of saline or laminarin, a β-1,3-glucan derived from the cell wall of the brown algae *Laminaria digitata*. Proteome mapping of the total hemocyte count through NanoLC-MS/MS was performed, and the proteomes of five saline-, five laminarin-injected and five uninjected individuals were compared. This approach was chosen as a means to characterize an early hemocyte response to β-1, 3-glucan, as well as to show the effects achieved by injection of saline alone since this treatment is widely used as a control for injection experiments in crustaceans. Here, we show clearly that such an approach is problematic when studying expression in hemocytes if not uninjected animals are included as a comparison.

**Figure 7:**
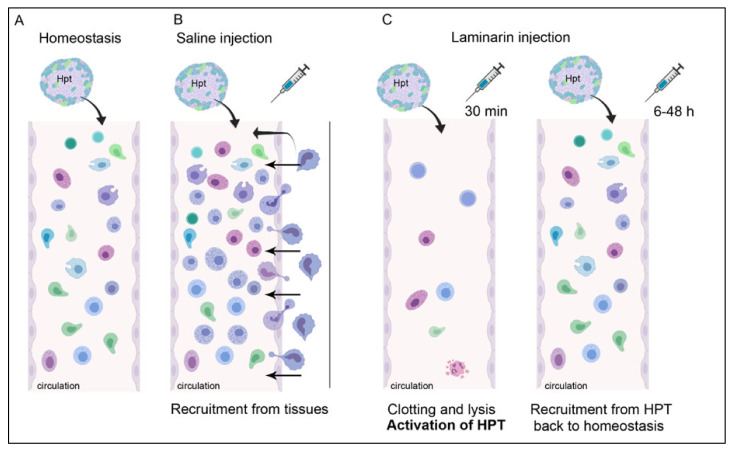
Illustration showing the events in crayfish peripheral circulation after injection of saline or laminarin. (**A**) Normal cell homeostasis in uninjected crayfish. (**B**) Saline injection results in a rapid recruitment of hemocytes, mainly mature GCs from surrounding tissues. (**C**) Laminarin injection causes a dramatic loss of hemocytes due to clotting, lysis and encapsulation of foreign glucan molecules. At the same time cytokines are released (i.e., astakine 1) into the plasma and induce high activity in the hematopoietic tissue (HPT) with release of new cells and also increased proliferation as we have earlier shown [22]. This figure was created with BioRender.com.

Changes in hemocyte proteins have been reported in three closely related crustaceans after injection of WSSV, compared to saline injection after 12 hours [43], or after 24 h [27,44]. When comparing the results of these studies it is obvious that they differ to a large extent, with some proteins upregulated in one study but downregulated in another. These types of inconsistencies make it difficult to draw conclusions about the immune response based upon the literature. A major problem is the variation between individuals occurring among crustacean species, as is shown by the high variation in total hemocyte count in our experiments. In related studies in other crustaceans, no information about different hemocyte types in the different treatments is considered. There is a dramatic variation in THC between animals [45], and in order to correctly test effects of a treatment, each individual has to be analyzed before and after treatment. By analyzing THC in this way, we show here (Figure 1a), that laminarin injection leads to a decrease in THC, while saline can induce an increase. We also showed (Figure 1c) that the response of saline injection and laminarin injection on total hemocyte number over time is totally different with a rapid increase after saline injection and the opposite when laminarin dissolved in saline is injected. This is a well-known fact that has been shown earlier for crayfish [22,35]. This is an important reason why it is difficult to compare results from an injection of for example WSSV with a saline injection, and why differences are not consistent between different publications. Another observation after injection of saline or laminarin, was an increase in the proportion of granular hemocytes. After saline injection, this is most likely due to rapid recruitment of GC from tissues infiltrated by these cells and it was clear from the biomarker result that the most abundant and upregulated proteins in response to saline were characteristic of vesicle contents of mature GC, such as the SPH1, VMO1, Crustin1 and MBL [36]. When laminarin is injected, on the other hand, the increase in GC proportion is probably because SGCs are more susceptible to damage and are consumed to a greater extent than GCs, and that there is a recruitment of newly synthesized cells from the hematopoietic tissue which do not express these vesicle proteins to the same degree as mature GCs [22]. See Figure 7 for a hypothetical scenario of the different injections. In summary, we show that comparing expression in hemocytes after an injection with a pathogen or pathogen-associated molecule and only use a vehicle control is problematic. For other tissues, this would work since these are stable in cell composition.

One aim of our study was to find putative proteins which are involved in a response to fungal infection. In comparison with an uninjected control we detected only six proteins (Table 3), three of these were also detected when compared to saline injection and thus not specific for a β-1,3,-glucan. One of these was an i-type lysozyme, the main function of which is in anti-bacterial defense, where this enzyme hydrolyzes the β-(1,4)-glycosidic bond in the bacterial cell wall component peptidoglycan [46]. Most lysozymes found in the animal kingdom are divided into c-type (chicken-type), g-type (goose-type) and i-type (invertebrate-type), based on amino acid sequence, biochemistry, and enzymatic properties [46]. A c-type lysozyme was also detected as upregulated after WSSV injection in *F. chinensis* [44], however in that case no uninjected control was tested so maybe this lysozyme was a general response to injection as the lysozyme in our study. The other two proteins which we detected in both saline and laminarin injected crayfish were Masquerade and Crustin 1 antimicrobial peptide. Masquerade is an opsonic protein and can recognize different microbial cell wall components [39], whereas crustins are peptides with broad antimicrobial activity [47]. As is elegantly shown by Ramond et al. [48] in *Drosophila melanogaster* larvae, a high number of genes are changed in expression in plasmatocytes only 45 min after a clean injury. Among these were several different antimicrobial peptides, and lysozyme-like pattern-recognition proteins (PGRPs). We have not shown if masquerade, crustin and lysozyme are induced by injury only in this study, but our results still suggest that the upregulation of these proteins is some general response to injections. Several of the most upregulated proteins after saline injection are indications of a stress response, for example, glutathione peroxidase (oxidative stress), the heat shock cognate protein, and similar proteins were also detected in *Drosophila* [48]. Thus, saline injection most likely results in both a stress response and at the same time recruitment of mainly GCs from tissues to the circulation.

Three proteins were detected as candidates for a specific β-1,3,-glucan response, since these were not detected as highly expressed after saline injection or in uninjected crayfish. None of these have been characterized before in *P. leniusculus* and could be of interest for further study as putative antifungal molecules. One was a Kazal-type protease inhibitor. Members of the Kazal-type PI (KPI) family inhibit various serine proteinases, and have one or more Kazal domains, characterized by six conserved cysteine residues that form three intra-domain disulphide cross-links [49]. They are among the most abundantly expressed proteins in crustacean hemocytes. The first KPI to be reported from arthropod hemolymph was from *P. leniusculus* [50], and at least 26 Kazal domains have been detected in this species, with a high degree of sequence polymorphism [37,51]. KPIs are thought to be involved in invertebrate antimicrobial defense and have been shown to be up-regulated in response to WSSV in *F. chinensis*, and *Vibrio anguillarum* in the scallop *Chlamys farreri* [52,53]. Bacteriostatic activities of KPIs have been reported from Black tiger shrimp *Penaeus monodon*, *Hydra magnipapillata*, and jellyfish *Cyanea capillata*, from which the identified KPI, CcKPI1, also displayed antifungal activity [54,55,56]. A KPI from *P. leniusculus* was found to inhibit proteases released by the crayfish pathogen *Aphanomyces astaci*, an oomycete [51]. The identified glycine-rich peptide doesn’t resemble any previously described peptide in *P. leniusculus*, and a BLAST search doesn’t yield any results. The available sequence shows similarities to Procambarin, a peptide identified from hemocytes of *Procambarus clarkii* [57]. Glycine-rich peptides described in other species, such as *Scylla paramamosain*, *Acanthoscurria gomesiana*, *Armadillidium vulgare* and *Tityus serrulatus*, generally exhibit broad and potent antimicrobial activity [58,59,60,61] The putative chitin-binding protein is another interesting find, as it could be a fungal infection-specific immune protein, since chitin is a common component of fungal cell walls. However, without further characterization, it is difficult to judge the specific functions of this protein.

An interesting observation when we compared laminarin and saline-injected crayfish was that the most abundant and highly upregulated proteins were three large plasma proteins, the clotting protein, α2M and βGBP. We interpret that this result means there are cells that bind to these proteins, and possibly such cell types are common among those released from the hematopoietic tissue. We have previously shown that the clotting protein binds to cells in the hematopoietic tissue, and therefore it is likely that some of these cells expose a receptor for this protein on their surface [62]. A receptor for βGBP has also been identified in hemocyte membranes [6]. In *Penaeus monodon* α2M was demonstrated to be upregulated after a *Vibrio* infection [63]. Furthermore, it was shown that one of the domains in α2M can bind to transglutaminase [63], the enzyme responsible for polymerization of the clotting protein [3]. Taken together, it is likely that these three plasma proteins are all involved in the response to laminarin, and probably help the hemocytes to encapsulate the glucan (or a fungus) by binding to the cells in a network of clot.

One protein that was further detected with high fold change in the laminarin injected crayfish in comparison with saline or uninjected controls was α-actinin. This protein is a ubiquitous protein in all cells, where it is a part of the cytoskeletal structure. Its main function is to bind actin, the protein that makes up the cytoskeletal filaments, by forming cross-links between the actin filaments [64]. One possible explanation for this high fold change of α-actinin is that it is a sign of broken cells and attachment on cell surfaces. Studies in *Drosophila* have suggested that extracellular α-actinin could have an additional function, by acting as a damage-associated molecular pattern (DAMP) [65,66]. DAMPs are molecules normally present in the cell, that are released into the extracellular space by damaged and dying cells, there acting as signalers to promote inflammation and tissue repair [67,68], and may therefore be a result from wounding.

When we compared mRNA levels of the identified upregulated proteins in Table 3 between laminarin-injected, saline-injected, and untreated individuals using semi-quantitative RT-PCR, the results showed that the genes were constitutively expressed in all individuals. However, after testing mRNA expression by qRT-PCR before and after injection individually, we could only find a slight increase in the expression of the Kazal proteinase inhibitor, but not for the other transcripts. However, induction of mRNA may be transient and to be certain if there is a β-1,3-glucan induction at transcription level, protein level or only by the presence of different cell types a further time study is needed for any putative β-1,3-glucan responsive protein.

The results of this study highlight the challenges of making comparisons in genetically diverse crustacean populations, with a high degree of individual variability when it comes to hemocyte numbers and composition. Several studies about crustacean host defense reactions towards various pathogenic microorganisms include injection of the pathogen into the animal followed by analyses of differential gene or protein expression. In most studies, no uninjected controls are included, and no information about hemocyte count (total or differential) is provided. Moreover, very few transcriptomic or proteomic studies of crustaceans include data about individual variations or even statistical significance (one exception shown in [43]), and many studies are done on pooled hemocyte samples. It is clear from *Drosophila* that injury by a needle awakens a severe response [48]. However, in contrast to *Drosophila* where well-characterized genetic strains in exact similar developmental stages (at the hour after hatching of a larva), and one specific cell type is used, studies in decapod crustaceans cannot be performed with such accuracy. The individuals are genetically diverse, their stage of development and their hemocyte population composition vary tremendously, and there are up until now only a few markers for different subpopulations of hemocytes. In order to find important immunological factors, there is a need for more detailed knowledge about the hemocyte subpopulations, changes in hemocyte composition after a treatment, as well as deep analysis of the function of putative up-or downregulated proteins and transcripts. It is clear, that in order to evaluate any putative up- or downregulation at mRNA or protein level there is an urgent need for more knowledge about hemocyte subpopulations so that sorting and analysis can be performed in similarity with the recent study in *Drosophila* [48].

In conclusion, we here show that an injection with saline and injection with a β-1,3,-glucan result in two completely different hemocyte populations in the circulation at least for two days after the injection, and thus this has to be considered when studying early expression differences in live crayfish and other crustaceans (Figure 7). We also show that recruitment of cells from tissues after saline challenge mainly increases the population of mature granular hemocytes. Furthermore, we found indication that a glycine-rich peptide, a Kazal-type protease inhibitor and a chitin-binding protein may be involved in response to a β-1,3,-glucan and thus a fungal infection, and also that the clotting protein, α2M and βGBP are likely to bind to cell surfaces as a response to injection of a β-1,3,-glucan and likely helps in an encapsulation reaction.

## 4. Materials and Methods

### 4.1. Animals

Freshwater crayfish, *P. leniusculus*, were obtained from Lake Erken, Sweden. The crayfish were maintained in tanks with aeration at about 10 °C. Apparently healthy and intermolt male crayfish of similar size were used for the experiments. Five laminarin-injected, five saline injected and five untreated crayfish were used for hemocyte preparation and further individual proteome analysis.

### 4.2. Injections and Cell Preparation

100 μL of 5 mg/mL laminarin dissolved in crayfish saline, or crayfish saline alone (CFS, 0.2 M NaCl, 5.4 mM KCl, 10 mM CaCl_2_, 2.6 mM MgCl_2_, 2 mM NaHCO_3_, pH 6.8) was injected into the base of a walking leg of the crayfish using a 23-gauge (G) needle (BD microlane). After six hours the hemocytes were collected as follows: 2 mL hemolymph was collected in a 1:1 volume with anti-coagulant solution (0.14 M NaCl, 0.1 M glucose, 30 mM trisodium citrate, 26 mM citric acid, 10 mM EDTA, pH 4.6 [11]) using a 18 G needle (BD microlane). Samples were centrifuged for 5 min at 800× *g*. The supernatant was discarded, and the cell pellet washed in 2 mL 0.15 M NaCl. The washing was repeated two more times. The cell number was determined with a hemocytometer. After the final washing step, the supernatant was removed and the hemocyte pellet stored at −80 °C, prior to the proteomic analysis. Five animals were injected with laminarin or saline and the hemocytes from each individual animal were separately saved and their total proteomes were analyzed individually as described below. Hemocytes from five non-treated crayfish were collected and used as naïve controls. In addition, total and differential hemocyte counts was analyzed in five individuals for each of the following treatment; uninjected, laminarin injection or saline injection after 2 and 6 h respectively. Statistical analyses for the hemocyte counts were performed with one-way ANOVA (with Fisher LSD test). In addition, hemolymph from individual crayfish were collected three days before any injection for a base level of hemocyte counts as above, and then laminarin or saline was injected and hemocyte counts were determined after six hours. Changes in hemocyte number were calculated as THC after injection divided by THC before injection. Statistical analyses were performed using a Mann–Whitney test, and one sample as identified as an outlier with the ROUT method.

### 4.3. Proteomic Analysis

Proteomic analysis was performed of the cell samples (five laminarin-injected, five saline-injected and five uninjected controls) at the mass spectrometry-based proteomics facility at Uppsala University.

#### 4.3.1. Chemicals and Reagents

Acetonitrile (ACN), acetic acid (HAc) and formic acid (FA) were obtained from Merck (Darmstadt, Germany). Protease inhibitor cocktail, phosphate-buffered saline (PBS), trifluoroacetic acid (TFA) n-octyl-β-D-glucopyranoside and ammonium bicarbonate (NH_4_HCO_3_) were purchased from Sigma Aldrich (St. Louis, MO, USA). For tryptic digestion, iodoacetamide (IAA), urea and dithiothreitol (DTT) were obtained from Sigma Aldrich and trypsin (Mass spectrometry grade; Promega, Mannheim, Germany) were used. Ultrapure water was prepared by Milli-Q water purification system (Millipore, Bedford, MA, USA).

#### 4.3.2. Protein Extraction and Quantification

The cell samples were lysed in 130 µL of lysis buffer (6 M urea and PBS containing 1% β-octyl glucopyranoside). Protease inhibitor cocktail (10 µL) was added during the sample preparation to prevent protein degradation. After homogenization, the samples were incubated for 90 min at 4 °C during mild agitation. The lysates were clarified by centrifugation for 30 min (10,000× *g* at 4 °C). The supernatant containing extracted proteins was collected and further processed.

The total protein content in the samples was determined using the DC Protein Assay Kit (BioRad Laboratories, Hercules, CA, USA), which is based on the modified Lowry method with bovine serum albumin as standard. The DC assay was carried out according to the manufacturer’s instructions using 96-well microtiter plate reader model 680 (BioRad Laboratories).

#### 4.3.3. On-Filter Tryptic Digestion of Proteins

Aliquots corresponding to 35 μg of proteins were used for digestion. An on-filter digestion protocol was used for tryptic digestion of the samples using 3 kDa filters (Millipore, Ireland). Centrifugation was carried out at a centrifugal force of 14,000 g throughout the protocol. A volume of 10 μL of 45 mM aqueous DTT was added to all samples and the mixtures were incubated at 50 °C for 15 min to reduce the disulfide bridges. The samples were cooled down to room temperature and 10 μL of 100 mM aqueous IAA was added before incubating the mixtures for an additional 15 min at room temperature in darkness to carabamido methylate the cysteines. The samples were transferred to spin filters that had been pre-washed with 250 µL of 20% acetonitrile (ACN) for 15 min and then 500 µL of water for 20 min. Next, the samples were centrifuged for 10 min to remove the added salts, detergents and other interfering substances. An additional volume of 200 µL of 50 mM NH_4_HCO_3_ in 20% ACN was added and the filters were spun for 15 min followed by 150 µL of 50 mM NH_4_HCO_3_, and centrifugation for another 10 min. Finally, a volume of 100 µL of 50 mM NH_4_HCO_3_ and 17 µL of trypsin (0.1 µg/µL) was added to the samples. The tryptic digestion was performed at 37 °C overnight in darkness. The digests were spun through the filter for 20 min to collect the tryptic peptides. An additional volume of 100 µL of 20% ACN, 1% acetic acid was added and the filters were spun for 10 min and pooled with the first tryptic peptide filtrate. The collected filtrates were vacuum centrifuged to dryness using a SpeedVac system ISS110 (Thermo Scientific, Waltham, MA, USA).

#### 4.3.4. NanoLC-MS/MS for Protein Identification

The samples were analyzed using a QExactive Plus Orbitrap mass spectrometer (Thermo Fisher Scientific, Bremen, Germany) equipped with a nano-electrospray ion source. The peptides were separated by reversed-phase liquid chromatography using an EASY-nLC 1000 system (Thermo Fisher Scientific). A set-up of pre-column and analytical column was used. The pre-column was a 2 cm EASY-column (1D 100 µm, 5 µm C18) (Thermo Fisher Scientific) while the analytical column was a 10 cm EASY-column (ID 75 µm, 3 µm, C18; Thermo Fisher Scientific). Peptides were eluted with a 150 min linear gradient from 4% to 100% acetonitrile at 250 nL min/1. The mass spectrometer was operated in positive ion mode acquiring a survey mass spectrum with resolving power 70,000 (full width half maximum), m/z = 400–1750 using an automatic gain control (AGC) target of 3 × 10^6^. The 10 most intense ions were selected for higher-energy collisional dissociation (HCD) fragmentation (25% normalized collision energy) and MS/MS spectra were generated with an AGC target of 5 × 10^5^ at a resolution of 17,500. The mass spectrometer worked in data-dependent mode.

#### 4.3.5. Mass Spectrometry Data Handling

The acquired data (RAW-files) were processed in MaxQuant 1.5.1.2 (18) and database searches were performed using the implemented Andromeda search engine. MS/MS spectra were correlated to FASTA databases containing proteins from crustaceans extracted from the UniProt database (release November 2019) and transcriptome database for *P. leniusculus* (BioProject PRJNA259594), TSA: GBYW01000001:GBYW01075939. A decoy search database, including common contaminants and a reverse database, was used to estimate the identification false discovery rate (FDR). An FDR of 1% was accepted. The search parameters included: maximum 10 ppm and 0.6 Da error tolerances for the survey scan and MS/MS analysis, respectively; enzyme specificity was trypsin; maximum one missed cleavage site allowed; cysteine carbamido methylation was set as static modification and oxidation (M) was set as variable modification. The search criteria for protein identification were set to at least two matching peptides. Label-free quantification was applied for comparative proteomics.

### 4.4. Qualitative Data Analysis

For the quantitative analysis all RAW-data files were quantitatively analyzed by the quantification software MaxQuant 1.5.1.2. Protein identification was performed by a search against the same database as for the qualitative analysis. The results of all samples were combined to a total label-free intensity analysis for each sample to get LFQ values (see details in Supplementary Table S1). Further normalization by subtracting the average of the raw LFQ values and log2 transformation was performed by using the GiaPronto software [69]. Proteins present in all samples were further analyzed. For pairwise comparisons between each treatment comparison, two-tailed Student’s t-tests (assuming equal variances) were performed on the generated full protein expression list and a *p*-value < 0.05 was considered as statistically significant. To identify differentially expressed proteins, normalized LFQ values from the two treatments based on their log2 ration and associated *p*-values were plotted in a Volcano plot using GiaPronto and a limit for fold change as Log2ratio > 0.58, and *p* (−log) >5. Proteins that were both abundant and upregulated in one of the treatments were assigned after multiplying the log2 ratio with the abundance as biomarkers [69].

### 4.5. RNA Extraction and mRNA Expression Analysis

The mRNA expression of four of the upregulated proteins (Table 3) was evaluated with RT-PCR. To compare expression on a transcript level, total RNA was extracted from the hemocytes of five crayfish from each group, which had been injected with laminarin, saline, or were untreated. Crayfish were injected and hemocytes collected as was described for samples sent for proteomic analysis. RNA was extracted and cDNA synthesized as follows. Briefly, hemocytes were homogenized in Trizol (Thermo Fisher), and RNA extracted with chloroform (Supelco). After centrifugation, the aqueous phase was transferred to a new tube. RNA was precipitated with 2-Propanol (Supelco) overnight, after which the sample was centrifuged and the 2-propanol removed. RNA pellet was washed twice with 75% ethanol and allowed to dry before being dissolved in 10µL Dnase/Rnase-free waster (Thermo Fisher), and then cDNA was synthesized using the Primescript cDNA synthesis kit (Takara) according to the manufacturer’s instructions, with oligo dT primers. The PCR reaction for each gene was prepared in 50 µL, using Phusion^TM^ High Fidelity DNA Polymerase (Thermo Fisher), with the 5X Phusion^TM^ HF Buffer and dNTP Mix provided by the manufacturer, according to their instructions. The PCR program was 98 °C for 30 s, followed by 30 cycles of 98 °C for 30 s, 58 °C for 30 s, and 72 °C for 45 s, and one cycle of 72 °C for 5 min. The PCR products were separated on a 1.5% agarose gel stained with SYBR^TM^ Safe DNA Gel Stain (Invitrogen) and visualized with FujiFilm Life Science Imaging Systems LAS4000, and the bands were then analyzed using ImageJ.

The expression of the identified Kazal-type proteinase inhibitor, the i-type lysozyme, masquerade and a putative chitin-binding protein was further evaluated by qRT-PCR in individual crayfish before and after injection of laminarin or saline (for primer sequences see Supplementary Table S4). Then, 0.75 mL hemolymph for RNA extraction was collected in anticoagulant from naïve crayfish as previously described, and hemocytes were then pelleted and stored in TRIzol (Thermo Fisher) at −80 °C. The animals were then allowed to recover for three days and then injected with 0.1 mL laminarin or CFS as previously described. After 6 h, 0.75 mL hemolymph was collected again, and hemocytes were stored the same way. For RNA extraction, the samples were thawed on ice and homogenized with a tissue grinder and by passing through a 0.4 mm needle. Total RNA was extracted using the PureLink RNA Mini kit (Thermo Fisher), according to the manufacturer’s instructions., and 0.21 µg RNA from each sample was used for cDNA synthesis, using the PrimeScript 1st strand cDNA Synthesis Kit (Takara). The cDNA was stored at −20 °C until use. The 18S gene transcript was used as an internal control for each sample (for primer sequences see Supplementary Table S2). A standard curve was prepared for each set of primers by mixing equal amounts of cDNA from each sample, and preparing a dilution series (10^−1^, 10^−2^, 10^−3^, 10^−4^), and also including a dilution of 5*10^−1^. qPCR was performed in 25 µL reactions using the QuantiTect SYBR^®^ Green PCR Kit (Qiagen), each reaction containing 12.5 μL QuantiTect SYBR Green PCR Master Mix, 1.5 μL forward primer (0.5 mM), 1.5 μL reverse primer (0.5 mM), 4.5 μL RNase-Free Water, and 5 μL of standard curve dilution or 5 μL 1:10 dilution of cDNA samples. Each sample was performed in duplicates. RNase-Free Water was used as negative controls, and non-template controls were included. PCR amplification was performed as follows: 15 min at 95 °C, 40 cycles of 15 s at 94 °C, 30 s at 58 °C and 30 s at 72 °C. The results were analyzed as a change in relative expression in hemocytes from one individual animal, six h after injection compared to the expression before in each individual crayfish (relative expression after injection/relative expression before injection). Statistical analyses were performed with a one sample t- and Wilcoxon test.

## Figures and Tables

**Table 1:** The twelve most upregulated proteins after saline injection. Accession number in the transcriptome database for *P. leniusculus* (BioProject PRJNA259594), TSA: GBYW01000001:GBYW01075939 is given, limit Fold change Log2ratio > 0.58, *p* (−log) >5.

Accession Number	Protein Name	Fold Change, Log2ratio	*p*-Value (−log)
GBYW01031372.1	Glutathione peroxidase 3 (GPX)	2.708	14.95
GBYW01022610.1 GBYW01021121.1 GBYW01005009.1	Heat shock cognate 71kDa protein	2.374	17.72
GBYW01026927.1	Trypsin-like serine protease	2.170	15.740
GBYW01024713.1	CLIP-domain serin protease	2.088	5.28
GBYW01017647.1 GBYW01041647.1	Flotillin-like protein	2.041	9.49
GBYW01021864.1	PDGF/VEGF domain protein	2.031	7.41
GBYW01011929.1	Peroxidase	1.994	8.12
GBYW01037257.1	Vitelline membrane outer layer protein 1 (VMO1)	1.884	7.35
GBYW01020841.1 GBYW01022391.1	Delta-1-pyrroline-5-carboxylate dehydrogenase	1.845	14.50
GBYW01031650.1	Unknown	1.694	6.34
GBYW01028873.1	i-type lysozyme	1.673	8.474
GBYW01030110.1 GBYW01030111.1	Unknown	1.617	8.11

**Table 2:** “Biomarkers” after saline injection. Biomarker value is calculated as the multiplication of abundance (mean for the treatment) with the fold change as Log2ratio). Accession number in the transcriptome database for *P. leniusculus* (BioProject PRJNA259594), TSA: GBYW01000001:GBYW01075939.

Accession Number	Protein Name	Biomarker Value	Fold Change Log2Ratio	*p*-Value
GBYW01037257.1	VMO1	6.656	1.8836	0.006
EF523612.1	Crustin 1	7.838	1.319	0.002
AY861652.1	SPH1	5.418	1.404	0.0005
GBYW01031372.1	Glutathione peroxidase (GPX)	1.767	2.708	3,15199E-05
AY861653.1	Mannose binding lectin	5.133	1.1447	0.003
Y11145.2	Masquerade	7.177	0.652	0.003
GBYW01028010.1	Kazal-type PI one domain	5.682	0.949	0.009
GBYW01031855.1	Kazal-type PI Agrin-like	6.029	0.823	0.022

**Table 3:** Specifically, upregulated proteins after laminarin injection compared to saline injection and uninjected controls, respectively. Accession number in the transcriptome database for *P. leniusculus* (BioProject PRJNA259594), TSA: GBYW01000001:GBYW01075939 is given, limit Fold change Log2ratio < 0.58, *p* (–log) >5.

Proteins Upregulated	Accession Number	Proteins Upregulated	Accession Number
In Laminarin and Saline Injection Compared to Uninjected		In Laminarin Compared to Uninjected	
i-type lysozyme	GBYW01028873.1	Glycine-rich peptide	GBYW01011676.1
Crustin -like AMP	GBYW01028095.1	Kazal-type	GBYW01031549.1
Masquerade	Y11145.2	Putative chitin binding protein	GBYW01037126.1

## Data Availability

Raw LFQ data is provided as Supplementary Table S1.

## Supplementary Materials

The following are available online at https://www.mdpi.com/article/10.3390/ijms22126464/s1, Table S1: LFQ intensity values, Table S2: Up- and downregulated proteins after saline injection. Table S3: Up- and downregulated proteins after laminarin injection. Table S4: Primer sequences used for RT-PCR analysis.

## Abbreviations

α2M	Alfa-2-macroglobulin
AMP	antimicrobial peptide
βGBP	Beta-1,3-glucan binding protein
proPO	prophenoloxidase
SPH	serine protease homologue
VMO1	vitelline membrane outer layer 1 homologue
