# Supplementary material for: Early Changes in Crayfish Hemocyte Proteins after Injection with a β-1,3-glucan, Compared to Saline Injected and Naive Animals
Source: Int J Mol Sci. 2021 Jun 16;22(12):6464. doi: 10.3390/ijms22126464 (PMC8234337; doi:10.3390/ijms22126464)
Supplement: Supplementary file 1 [file ijms-22-06464-s001.zip › Supplementary Table S4.pdf]

Table S4: Primer sequences used for RT-PCR analysis (F=forward, R=reverse).

Gene	Sequence 5′-3′
Kazal-type proteinase inhibitor F	GGTGAACTCACAGGTGTTGT
Kazal-type proteinase inhibitor R	ACTGTTCTCTGCTCCTCTGT
i-type lysozyme F	AGTGCCTGTTCAGTCAGCTC
i-type lysozyme R	GGAACCAG CCACAGTCTACC
i-type lysozyme F, qRT-PCR	CAGGAATATGCCCCTGTGGATGA
i-type lysozyme R, qRT-PCR	TTAGTGGATGCCAAGTTTAGCGC
Masquerade F	TAAACACAAGCGGAGTAGGC
Masquerade R	TAGCCACTACCACCTGTTCC
GBYW01037126.1 (putative chitin-binding) F	GAGTCACGGAGGATCGATTG
GBYW01037126.1 (putative chitin-binding) R	GAAGCCTGTTTCAGAGGTCC
18S ribosomal protein F	AGTTTCAGCACATCCTGCGT
18S ribosomal protein R	TCAGTCATCTCTCCAGCACG
18S ribosomal protein F, qRT-PCR	AGTTTCAGCACATCCTGCCTTCCCTTCAGA
18S ribosomal protein R, qRT-PCR	ATCTTCTCAACCTCTTCCTCAGTCATCTCTC
